# Ultrasonic misdiagnosis of giant pediatric testicular yolk sac tumor: A case report and literature review

**DOI:** 10.3389/fped.2022.1058037

**Published:** 2022-12-20

**Authors:** Zilong Wang, Fuding Lu, Changze Song, Xinkun Wang, Naifa Li, Jiawen Zhai, Baohong Jiang, Jianpeng Yuan, Zheng Yang, Xujun Xuan

**Affiliations:** ^1^ Department of Andrology, The Seventh Affiliated Hospital, Sun Yat-sen University, Shenzhen, China; ^2^ Department of Radiology, The Seventh Affiliated Hospital, Sun Yat-sen University, Shenzhen, China; ^3^ Department of Pathology, The Seventh Affiliated Hospital, Sun Yat-sen University, Shenzhen, China; ^4^ National Research Center for Assisted Reproductive Technology and Reproductive Genetics, Cheeloo College of Medicine, Shandong University, Jinan

**Keywords:** yolk sac tumor, ultrasonic misdiagnosis, AFP, BEP chemotherapy, orchidectomy, case report

## Abstract

**Background:**

Yolk sac tumor is the most common malignant nonseminomatous germ-cell tumor in children characterized by elevated level of *α*-fetoprotein (AFP), accounting for 70%–80% of all cases. However, giant yolk sac tumors that involve the entire testicle may be misdiagnosed by color Doppler ultrasonography as orchitis. Therefore, we described a case of a 2-year-old pediatric patient with a giant testicular yolk sac tumor that was misdiagnosed by ultrasonography as orchitis, in order to evaluate the role of measuring AFP levels in the initial diagnosis to aid in the accuracy of the definitive diagnosis of testicular yolk sac tumor.

**Case presentation:**

A 2-year-old boy received outpatient visits for unintentional swelling of the right scrotum for 7 days. Physical examination showed a rubbery swelling of the right scrotum with rejective touch. Then, the patient underwent perineal color Doppler ultrasonography in outpatient visits. The result showed a right testicle size of 29 mm × 22 mm × 20 mm with heterogeneous echogenicity and abundant blood ﬂow, supporting the initial diagnosis of orchitis. However, the initial surgeon was skeptical of the ultrasonography diagnosis. Thus, the patient was admitted to the Department of Andrology on day 2 for further serological and imaging examination. The serum AFP level on day 3 was 323.77 ng/ml. The results of CT and MRI showed a giant tumor of the right testis (26 mm × 21 mm × 29.6 mm) with multiple lymphoid hyperplasia in the inguinal region bilaterally. The patient received radical orchidectomy without lymph node dissection on day 9. The results of postoperative pathological examination confirmed giant testicular yolk sac tumor (T1N0M0S1, Stage Is) and was positive for AFP and SALL4 in immunohistochemistry staining. The patient received three courses of bleomycin–etoposide–cisplatin chemotherapy in the Department of Pediatrics after multidisciplinary team meeting on postoperative days 14, 37, and 58, respectively. During chemotherapy and follow-up, the patient's AFP and lactate dehydrogenase levels continued to decline, and eventually remained within normal range on postoperative day 84.

**Conclusion:**

Measuring the AFP level was necessary for initial diagnosis and follow-up in pediatric cases of testicular enlargement. Radical orchidectomy combined with postoperative bleomycin–etoposide–cisplatin adjuvant chemotherapy was an effective treatment strategy for pediatric giant testicular yolk sac tumors.

## Introduction

Yolk sac tumor (YST), which originates from the extraembryonic endoderm, is the most common malignant nonseminomatous germ-cell tumor (NSGCT) in children characterized by elevated level of *α*-fetoprotein (AFP) (1), accounting for 70%–80% of all cases (2). However, due to abundant blood ﬂow and similar values of peak systolic velocity (PSV) and resistive index (RI) (3), giant YSTs whose tumor-infiltrating region involving the entire testicle may be misdiagnosed as orchitis by color Doppler ultrasonography, with a prevalence of appropriately 30% (4). Therefore, for children with giant testicular YST, the accuracy of the initial diagnosis becomes critical.

Orchidectomy is the main treatment of choice for YST (5), but whether intraoperative lymph node dissection or postoperative bleomycin–etoposide–cisplatin (BEP) chemotherapy should be applied remains controversial, especially for children aged 2 years or younger (6). Although the overall survival (OS) rate of pediatric YST is greater than 90%, approximately 10%–30% of patients experience recurrences and distant metastasis within 2 years of initial treatment (7).

We described a case of a 2-year-old child with giant testicular YST that was misdiagnosed as orchitis by color Doppler ultrasonography. This patient received radical orchidectomy without lymph node dissection and postoperative BEP chemotherapy. The objective of this case report was to evaluate the role of measuring AFP levels in the initial diagnosis to aid in the accuracy of the definitive diagnosis of testicular yolk sac tumor.

## Case description

### Preoperative condition

**Figure 1:**
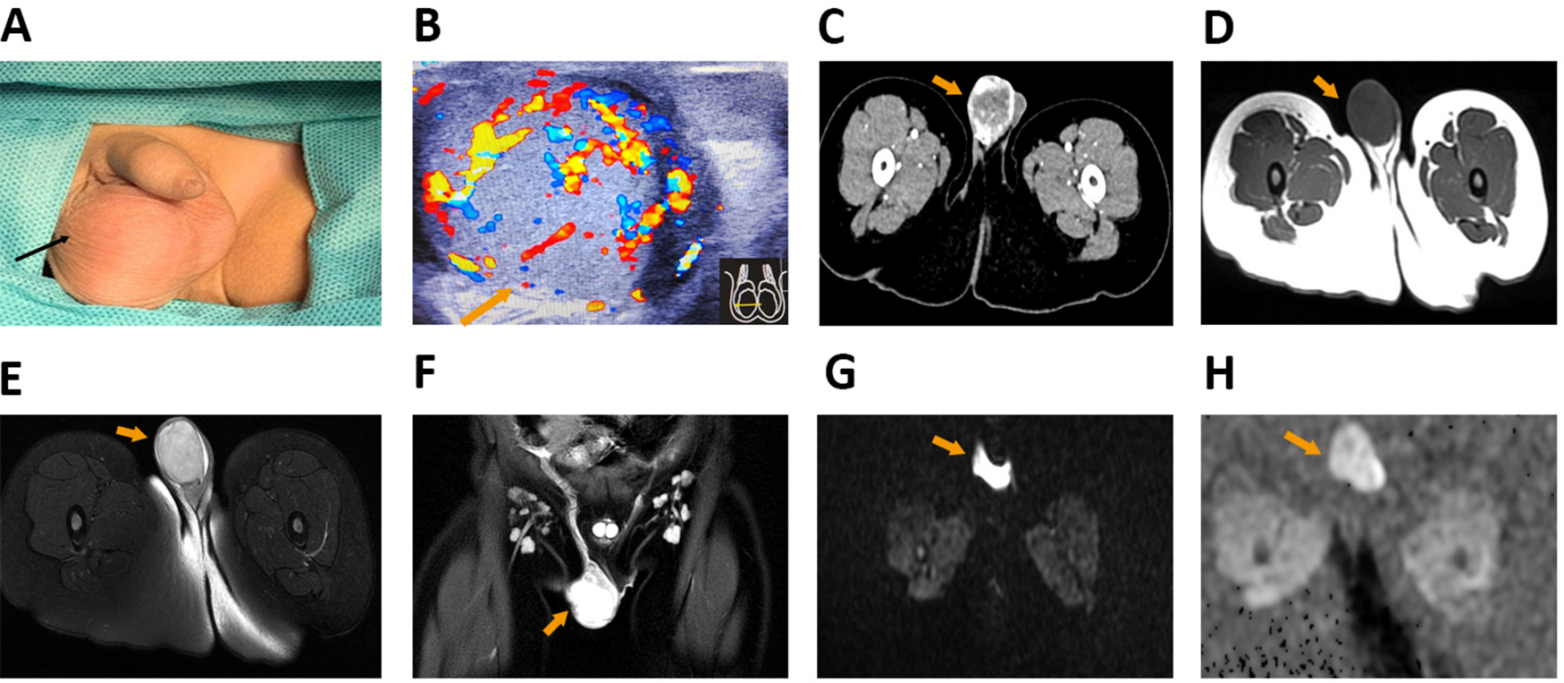
The results of preoperative examination. (**A**) The physical examination showed right scrotal painless and rubbery swelling. (**B**) The results of perineal color Doppler ultrasonography showed that the size of right testis was 29 mm × 22 mm × 20 mm, the PSV was 15.4 cm/s, and RI was 0.65. (**C**) CT showed the right testis with space-occupying lesions. (**D–H**) MRI showed slightly equal SI on T1WI (**D**), high SI (**E**) and homogenized enhancement of enlarged multiple lymph nodes in bilateral inguinal region (**F**) on T2WI, insignificant diffusion restriction on DWI (**G**), and high SI on ADC (**H**) in the lesion of right testis. PSV, peak systolic velocity; RI, resistive index; SI, signal intensity; T1WI, T1-weighted imaging; T2WI, T2-weighted imaging; DWI, diffusion-weighted imaging; ADC, apparent diffusion coefficient.

The 2-year-old boy received outpatient visits for an unintentional swelling of the right scrotum with rejective touch for 7 days on 1 March 2022 (day 1). The patient had no history of any perineal trauma, urinary infection, or other abnormalities. The patient also had no history of psychological, genetic, or other disorders and no family history of testicular tumor. The patient had no relevant interventions in the past. Physical examination showed a rubbery swelling of the right scrotum with rejective touch (Figure 1A). The routine blood WBC was 18.35 × 10^9^/L. Perineal color Doppler ultrasonography showed that the right testis was 29 mm × 22 mm × 20 mm in size with heterogeneous echogenicity and abundant blood ﬂow, and the PSV was 15.4 cm/s and RI was 0.65. The left testis was 14mm × 10mm × 8 mm in size. These results supported orchitis of the right testis (Figure 1B). However, based on the experience with diagnosis and treatment, the initial surgeon was skeptical of ultrasonic diagnosis.

To further detained serum and imaging examination to prevent ultrasonic misdiagnosis, the patient was admitted to our department on 2 March 2022 (day 2) for further examination. The serum biomarkers test results on day 3 were as follows: AFP 323.77 ng/ml (normal range <20.00 ng/ml), lactate dehydrogenase (LDH) 267U/L (normal range 120–250 U/L), chorionic gonadotropin (HCG) <2.39 mIU/ml, and carcinoembryonic antigen (CEA) 2.54 ng/ml (normal range <5.00 ng/ml). Computed tomography (CT) found an occupying lesion in the right testis (Figure 1C). Magnetic resonance imaging (MRI) showed slightly equal signal intensity (SI) on T1-weighted imaging (T1WI), high SI on T2WI, insignificant diffusion restriction on diffusion-weighted imaging (DWI), and high SI on apparent diffusion coefficient (ADC) (26 mm × 21 mm × 29.6 mm) in the lesion of right testis, and homogenized enhancement of multiple lymph nodes with bilateral enlargement in the inguinal region (Figures 1D–H). Preoperative diagnosis was a right testicular tumor with reactive lymphoid hyperplasia in the bilateral inguinal region.

### Operative condition and pathologic results

**Figure 2:**
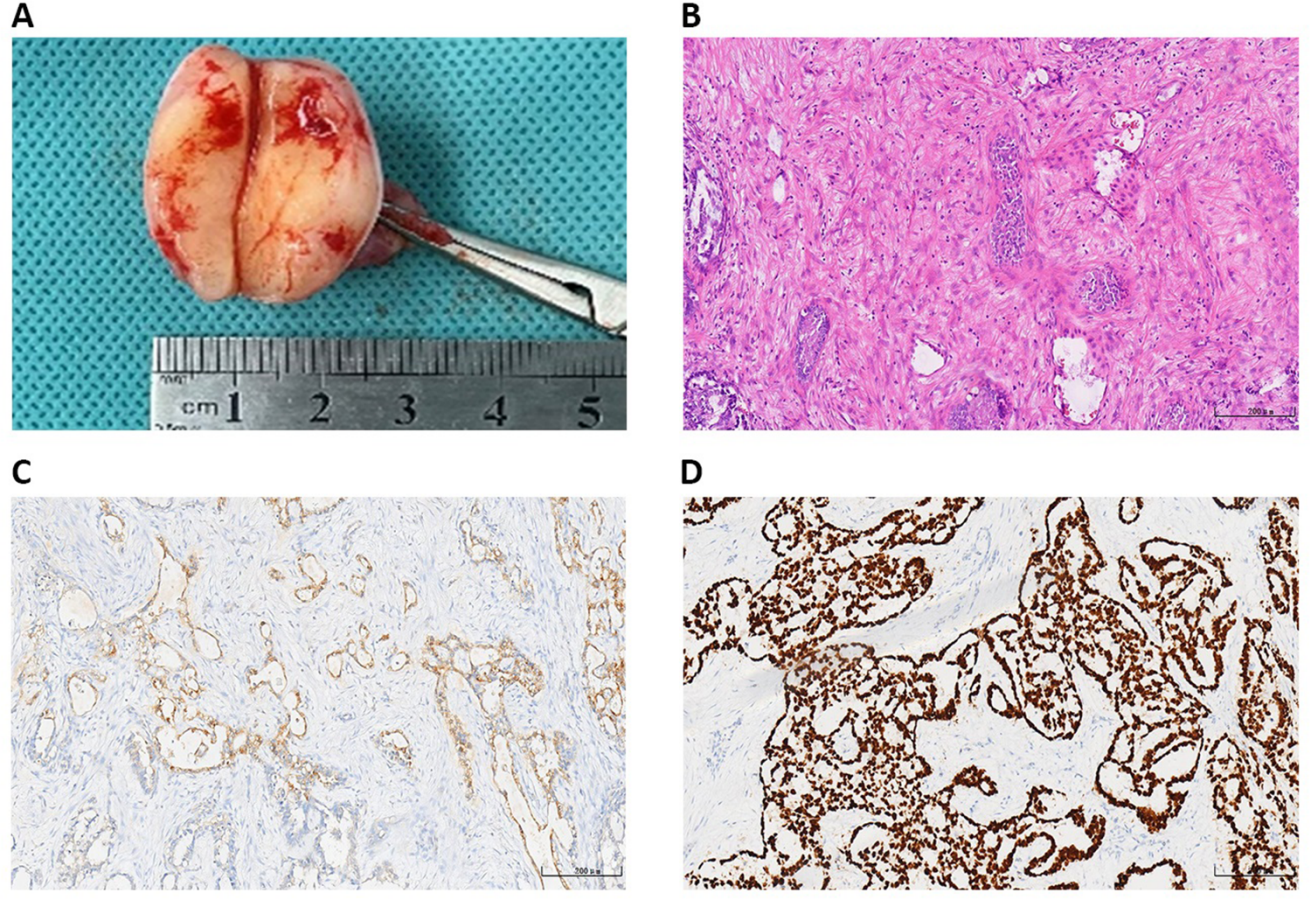
The postoperative pathology results. (**A**) Right testicular malignancy without capsular invasion. The results of postoperative pathology with HE staining (**B**) and AFP (+) (**C**), and SALL4 (+) (**D**) in IHC staining of testicular YST. AFP, *α*-fetoprotein; SALL4, spalt like transcription factor 4; IHC, immunohistochemistry; YST, yolk sac tumor.

The patient received radical orchidectomy on 9 March 2022 (day 9). We removed part of the tumor tissue and performed pathological examination to clarify the diagnosis, which confirmed the malignant tumor in the right testis without capsular invasion. Considering that the testicular capsule was in integrity and the inguinal lymph nodes were not the sentinel lymph nodes, we performed only radical orchidectomy without lymph node dissection in order to reduce surgical trauma. The postoperative pathology report confirmed that the right testicular YST did not invade the testicular capsule, spermatic cord, epididymis, and vascular system (Figures 2A,B). The results of immunohistochemistry (IHC) staining were positive for AFP, spalt like transcription factor 4 (SALL4), cytokeratin (CK), CD117, and placental alkaline phosphatase (PLAP), and negative for Vimentin, CEA, Oct3/4, and CD30 (Figures 2C,D and Supplementary Figures S1A–G). Postoperative diagnosis was right testicular YST (T1N0M0S1, Stage Is).

### Postoperative chemotherapy and prognosis condition

**Figure 3:**
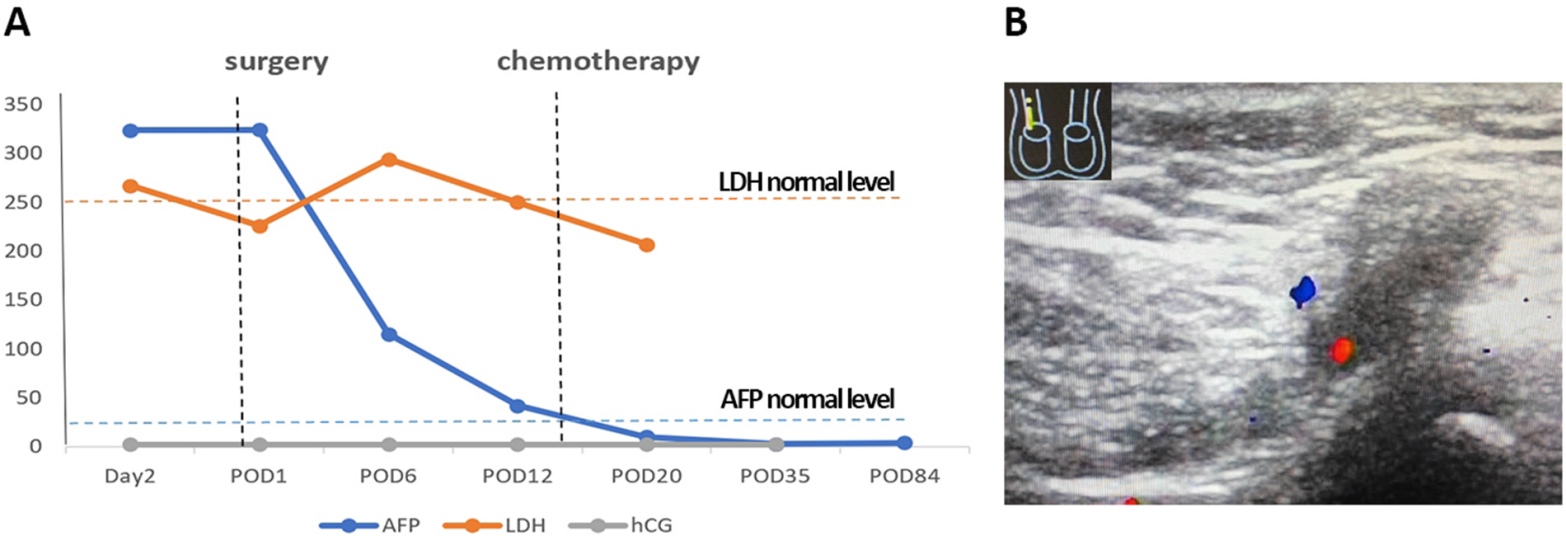
The results of postoperative reexamination. (**A**) The line graph of serum AFP, LDH, and HCG levels changes in the perioperative and chemotherapeutic period. (**B**) The results of reexamination of color Doppler ultrasonography showed hypoechoic and bloodless legion in right inguinal legion. POD, postoperative day. AFP, *α*-fetoprotein; LDH, lactate dehydrogenase; HCG, chorionic gonadotropin.

The AFP level of blood biomarkers continued to decrease on postoperative days 1, 6, and 12, from 324.05 ng/ml on 10 March (postoperative day 1) and 115.19 ng/ml on 15 March (postoperative day 6) to 41.94 ng/ml on 21 March (postoperative day 12). Despite increased fluctuations, the LDH level eventually decreased from 226 U/L on postoperative day 1 and 294U/L on postoperative day 6 to a normal level of 250 U/L on postoperative day 12. The HCG level was consistently below 2.39 mIU/ml within the normal range (Figure 3A). The patient’s condition was discussed in the multidisciplinary team (MDT) meeting, including Departments of Andrology, Pediatrics, Pathology, and Radiology. The decision was to give two to four courses of BEP chemotherapy, as a result of the large size of the tumor, bilateral inguinal lymph node enlargement without dissection, and high risk of recurrence.

**Figure 4:**
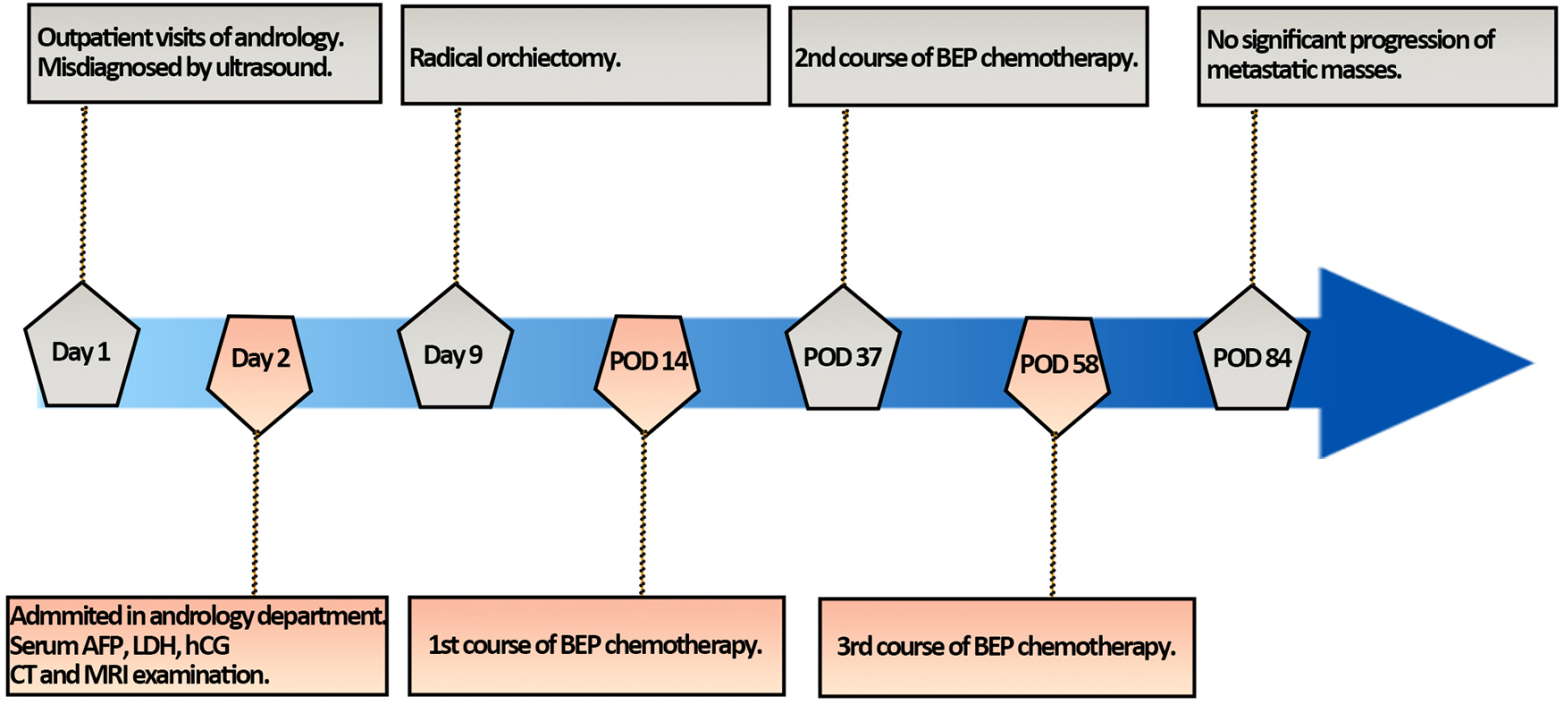
The flowchart of timeline for diagnosis and treatment process. POD, postoperative day.

The patient was transferred to the Department of Pediatrics for chemotherapy on 21 March 2022 (postoperative day 12). The patient received three courses of bleomycin (15 mg/m^2^ d1), etoposide (100 mg/m^2^ d1–5), and cisplatin (20 mg/m^2^ d1–5) chemotherapy without uncontrollable side effects on 23–27 March 2022 (postoperative days 14–18), 15–19 April 2022 (postoperative days 37–41), and 6–10 May 2022 (postoperative days 58–62), respectively. Within all the cycles of chemotherapy, the AFP levels remained within the normal range of 9.99 ng/ml on postoperative day 20 and 2.55 ng/ml on postoperative day 35 (Figure 3A). The LDH level was normal on postoperative day 20 with 207 U/L on postoperative day 35. During the 3-month chemotherapy and follow-up after radical orchidectomy, the patient had a good prognosis. On postoperative day 84, the AFP levels still remained within the normal range of 3.81 ng/ml (Figure 3A). The results of reexamination of color Doppler ultrasonography showed normal imaging of the perineal legion and the hypoechoic and bloodless legion of the right inguinal legion, with a left testis size of 10.6 mm × 8.0 mm × 6.3 mm (Figure 3B). The flowchart of the timeline for the diagnosis and treatment process schedule is shown in Figure 4.

## Discussion

This case report described a case of a 2-year-old boy with giant testicular YST that was misdiagnosed as orchitis by perineal color Doppler ultrasonography. Based on serum AFP levels and CT and MRI findings, the patient received radical orchidectomy without lymph node dissection. Postoperative pathological findings showed no involvement of the testicular capsule, spermatic cord, epididymis, and vascular system, and AFP (+) and SALL4 (+) on IHC, indicating that YST was of T1N0M0S1 and Stage Is. Considering the large size of the tumor and the high risk of recurrence without lymph node dissection due to bilateral inguinal lymphoid hyperplasia, the patient underwent three courses of BEP chemotherapy. Serum AFP levels continued to decline and the patient had a good prognosis during 3-month chemotherapy and follow-up after radical orchidectomy.

Appropriate and accurate preoperative examination procedures are crucial in screening for testicular YST in children. The main and initial clinical manifestation in children with testicular YST is painless enlargement of the testis (8). Color Doppler ultrasonography is the main screening procedure for perineal diseases, especially for pediatric testicular lesion (5, 9). However, it is difficult to use ultrasonography as a speciﬁc diagnostic tool for pediatric testicular YST because its tumor-infiltrating region involves the entire testicle. The Song’s study found a mean PSV of 12.9 cm/s, a mean RI of 0.54, and abundant blood ﬂow in testicular YST (3). In the present case report, the PSV was 15.4 cm/s and RI was 0.65, which were similar to the results of Song's study (3). However, there was a crossover between these parameters and normal reference values (10). It was difficult to use these parameters as specific diagnostic indicators for yolk sac tumors (3). Thus, this was the cause of diagnostic errors for this patient who was misdiagnosed by perineal color Doppler ultrasonography as orchitis. Because of the reasons outlined above, further appropriate biomarkers are needed for initial screening of children with testicular enlargement.

Elevated level of AFP is one of the most significant biomarkers in yolk sac tumors, accounting for more than 92% of all pediatric patients (11). The average percentage of AFP-positive YST tissues was higher than 80% (12). If a testicular mass and elevated AFP levels are present in a child older than 1 year, the patient may suffer from YST (4). In the present case report, the patient had abnormally elevated serum AFP level at preoperative examination, and postoperative pathology report confirmed right testicular YST rather than orchitis. Therefore, measuring the AFP level was necessary for initial diagnosis in a pediatric case of testicular enlargement. Meanwhile, after ultrasonography and serum tumor marker analysis, abdominal and retroperitoneal CT scans and MRI should be performed if there is concern that the lesion is malignant, especially for cases with elevated AFP levels.

Accurate assessment of lymph node metastasis is essential to surgical strategies and postoperative adjuvant therapy. The primary drainage site of the right testicular tumor, including YST, is the interaortocaval lymph nodes inferior to the renal vessels, followed by the paracaval and para-aortic nodes, and the pattern of retroperitoneal lymphatic drainage was from right to left (13). According to the preoperative CT and MRI findings, no abnormal interaortocaval lymph nodes were found in the patient. Meanwhile, retroperitoneal lymph node dissection (RPLND) has a minimal role in prepubertal testicular YST, as it usually presents with hematogenous spread to the lungs without retroperitoneal disease (14), and RPLND is only used in patients with a residual retroperitoneal mass or persistently elevated AFP after chemotherapy and orchidectomy (14). The patient underwent only radical orchidectomy without RPLND to reduce surgical trauma.

The efficacy of postoperative chemotherapy remains controversial. The recurrence rate in patients with lymph node invasion after initial chemotherapy had decreased from 30%–60% to 2%–3% (4). According to the guidelines for the treatment of testicular YST, only two courses of BEP chemotherapy are recommended for high-risk stage I patients (6). However, Geng et al. found that localized testicular YST patients without chemotherapy had a better prognosis because these patients were willing and able to be monitored (15). The patient received three courses of BEP chemotherapy after radical orchidectomy and had a good prognosis during 3-month chemotherapy and follow-up. Therefore, the confounding factors between management and prognosis need to be further investigated.

Elevated AFP level is a reliable marker for follow-up studies (16, 17), and stage I patients underwent a rigorous monitoring protocol that included chest and retroperitoneal imaging and tumor marker assessment after orchidectomy (14). The patient's AFP level decreased from 323.77 ng/ml preoperatively to 41.94 ng/ml on postoperative day 12, which was consistent with the accepted AFP half-life of 5 days (4). Moreover, the serum AFP levels remained within the normal ranges during and after chemotherapy, suggesting that radical orchidectomy with three courses of BEP chemotherapy are beneficial to giant testicular YST in children.

The following limitations exist in this study: First, we did not evaluate the effect of chemotherapy on the fertility of this 2-year-old child. Although the size of contralateral testis decreased after chemotherapy, it would be preferable to collect data on sex hormone levels and serum inhibin B, as well as routine semen analysis during and after adolescence to evaluate the reproductive capacity. Second, we did not evaluate whether the child should be applied to HCG to prevent possible infertility induced by chemotherapy in the future. The relationship between HCG injection and YST recurrence is uncertain. Finally, the patient should be in long-term follow-up to evaluate the prognosis and fertility after three courses of BEP chemotherapy following radical orchidectomy.

## Conclusion

Measuring the AFP level was necessary for initial diagnosis and follow-up in a pediatric case of testicular enlargement. Radical orchidectomy combined with postoperative BEP adjuvant chemotherapy was an effective treatment strategy for pediatric giant testicular YST.

## Data Availability

The original contributions presented in the study are included in the article/Supplementary Material, further inquiries can be directed to the corresponding author.
