# Supplementary material for: Ultrasonic misdiagnosis of giant pediatric testicular yolk sac tumor: A case report and literature review
Source: Front Pediatr. 2022 Dec 20;10:1058037. doi: 10.3389/fped.2022.1058037 (PMC9807905; doi:10.3389/fped.2022.1058037)
Supplement: Supplementary file 1 [file Image1.pdf]

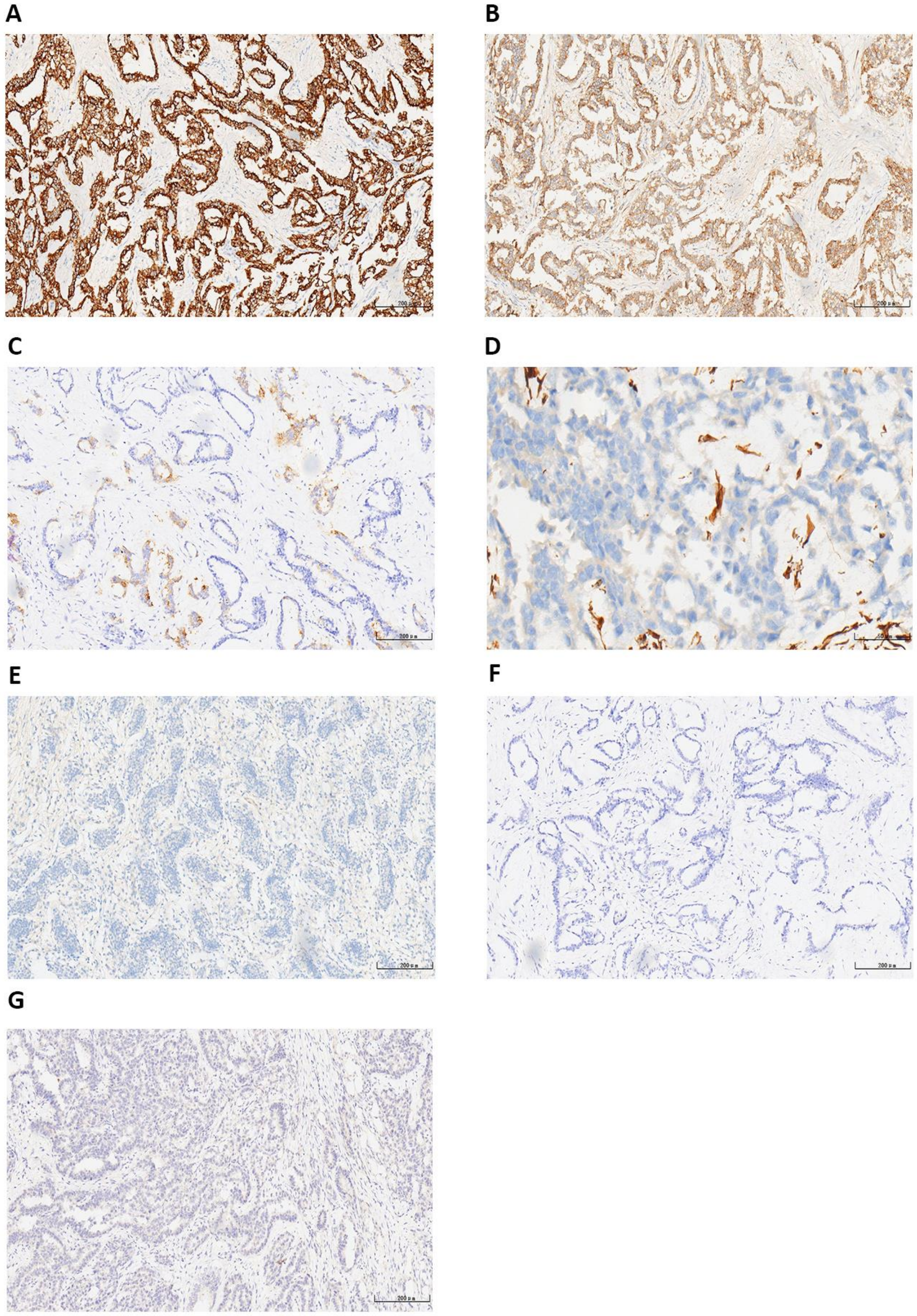

Supplementary Figure S1 The results of postoperative pathology in IHC staining of testicular YST. (A) CK(+), (B) CD117(+), (C) PLAP(+), (D) Vimentin(-), (E) CEA(-), (F)Oct3/4(-), (G) CD30(-).
